# Kinase Activity Profiling of Pneumococcal Pneumonia

**DOI:** 10.1371/journal.pone.0018519

**Published:** 2011-04-05

**Authors:** Arie J. Hoogendijk, Sander H. Diks, Tom van der Poll, Maikel P. Peppelenbosch, Catharina W. Wieland

**Affiliations:** 1 Center for Infection and Immunity Amsterdam, Academic Medical Center, University of Amsterdam, Amsterdam, The Netherlands; 2 Center for Experimental and Molecular Medicine, Academic Medical Center, University of Amsterdam, Amsterdam, The Netherlands; 3 Section Immunology, Department of Cell Biology, University Medical Center, Groningen, University of Groningen, Groningen, The Netherlands; 4 Department of Gastroenterology and Hepatology, Erasmus Medical Center, Rotterdam, The Netherlands; 5 Laboratory of Experimental Intensive Care and Anesthesiology, Academic Medical Center, University of Amsterdam, Amsterdam, The Netherlands; Facultad de Medicina, Uruguay

## Abstract

**Background:**

Pneumonia represents a major health burden. Previous work demonstrated that although the induction of inflammation is important for adequate host defense against pneumonia, an inability to regulate the host's inflammatory response within the lung later during infection can be detrimental. Intracellular signaling pathways commonly rely on activation of kinases, and kinases play an essential role in the regulation of the inflammatory response of immune cells.

**Methodology/Principal Findings:**

Pneumonia was induced in mice via intranasal instillation of *Streptococcus (S.) pneumoniae*. Kinomics peptide arrays, exhibiting 1024 specific consensus sequences for protein kinases, were used to produce a systems biology analysis of cellular kinase activity during the course of pneumonia. Several differences in kinase activity revealed by the arrays were validated in lung homogenates of individual mice using western blot. We identified cascades of activated kinases showing that chemotoxic stress and a T helper 1 response were induced during the course of pneumococcal pneumonia. In addition, our data point to a reduction in WNT activity in lungs of *S. pneumoniae* infected mice. Moreover, this study demonstrated a reduction in overall CDK activity implying alterations in cell cycle biology.

**Conclusions/Significance:**

This study utilizes systems biology to provide insight into the signaling events occurring during lung infection with the common cause of community acquired pneumonia, and may assist in identifying novel therapeutic targets in the treatment of bacterial pneumonia.

## Introduction

Due to its unique relationship with the environment, the lung must defend itself from infection by numerous inhaled micro-organisms. Although in general the lung is successful in doing so, bacterial pneumonia remains a major health burden. The Gram-positive bacterium *S. pneumoniae* is the main causative pathogen in community-acquired pneumonia (CAP), responsible for an estimated ten million deaths annually worldwide [1], [2], [3]. Increasing resistance of this common pathogen to antibiotics is a great concern [4], [5], [6].

Recognition of invading bacteria by the host is considered to occur mainly through toll-like receptors (TLRs). After interacting with their ligands, TLRs signal via adaptor proteins and kinases to ultimately activate Nuclear factor-κB (NF-κB) inducing inflammatory responses [7]. However, the interactions between bacteria and host cells are not confined to TLRs and ongoing intracellular signaling cascades may be much more extensive and complex than generally thought. Many studies on host pathogen interactions concentrate mainly on isolated pathways [8], [9], [10], [11]. Although elegant in emphasizing the importance of these single pathways, such studies do not address the synergy of the multitude of signal-cascades, activated upon recognition of pathogens. Systems biology provides tools to enable understanding of such complex matters.

Kinases comprise an important part of the intracellular responses mediated by a variety of receptors. Although it is highly likely that kinases mediate lung inflammation during pneumonia, knowledge about the activation of kinases during pneumonia is limited. Microarray-based kinome profiling approaches have been subject of development over the last years and an interesting tool to integral study signaling events [12], [13], [14]. Unraveling the complexities of the host-pathogen interactions during pneumococcal pneumonia can be of great value in finding new targets of therapy. Here we use a radio-kinome substrate array to determine kinase activities in the lungs during *S. pneumoniae* pneumonia in mice and furthermore attempt to elucidate complex interactions occurring during the course of the infection. To our surprise, we did not detect signaling pathways belonging to the TLR signaling cascades. In contrast, we detected pathways that induce chemotoxic stress and promoted the T helper 1 (Th1) response. In addition we found an overall reduction in WNT signaling. Canonical WNT signaling, named after the homology of WNT-genes with int-1 and wingless in Drosophila, is important in developmental signaling [15], [16]. However more roles of this signaling cascade have emerged (e.g. development of cancer)[17].

Moreover, we found a reduction in cell cycle activity during the course of *S. pneumoniae* pneumonia. This study is the first to apply kinome profiling using kinomics chip arrays in infectious diseases.

## Results

### Bacterial pneumonia

**Figure 1:**
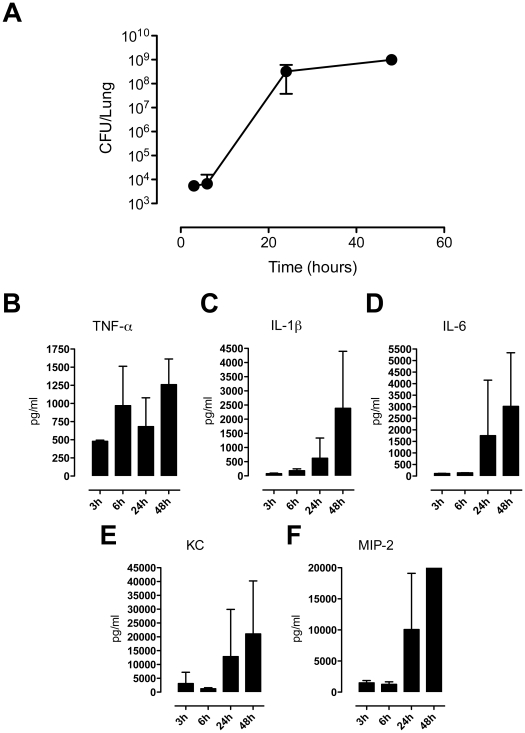
Bacterial growth and induction of cytokines and chemokines. Mice were inoculated with 5×10^4^ CFUs of *S. pneumoniae* via the intranasal route. Whole lung homogenates were harvested at the indicated time points. CFUs (a); cytokine (b–d) and chemokine (e,f) levels. Data are means ± SD of 3 mice per time point.

First, we determined the course of bacterial infection. After instillation of *S. pneumoniae* bacterial loads remained similar at 3 and 6 hours (Figure 1a). Between 6 and 24 hours bacterial loads in the lung increased exponentially (up to 5 logs increase). At this time an apparent maximum number of bacteria had been reached in the lung compartment, as no further increase was detected at 48 hours. The induction of lung inflammation was illustrated by increases in the pulmonary levels of all measured cytokines (Tumor necrosis factor-α (TNF- α), Interleukin (IL)-1β, IL-6) and chemokines (cytokine-induced neutrophil chemoattractant (KC), Macophage inflammatory protein (MIP)-2) during the course of bacterial pneumonia (Figures 1b–f).

### Kinome profile overview

**Figure 2:**
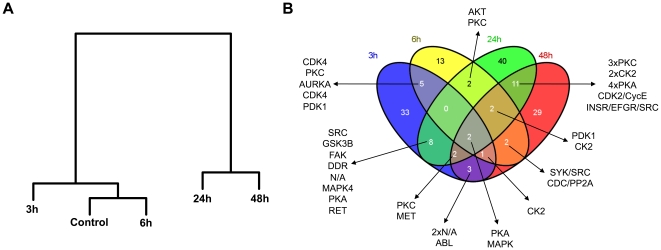
Clustering. Hierarchal clustering according to Johnsons of phosphorylation states of whole lung lysates [18] (a). Venn diagram of spots phosphorylated at measured time points. 3 hours is depicted in blue, 6 hours in yellow, 24 hours in green and 48 hours in red (b). Kinase activities spanning multiple time points (intermediate colors) are listed.

To determine the relation between each data-set of obtained kinome profiles we performed hierarchical clustering according to Johnson (Figure 2a) [18]. During *S. pneumoniae* infection the distance to the control increased throughout the course of infection and with increasing bacterial loads, indicating increased divergence from the initial kinase activity profile. Interestingly, 6 hours after infection, the kinome profile resembled that of the control more than the profile at 3 hours. This suggested that strong changes occurred in phosphorylation patterns early in infection. As the infection progressed more changes in the kinome profile were detected, creating a greater distance in the cluster. The greatest distance to the control was found at 24 and 48 hours, which cluster outside of the control and 3 and 6 hours group.

Overall, a total of 153 kinases were found to be activated significantly different from uninfected control lungs. In Figure 2b a multi-dimensional Venn diagram represents distribution of phosphorylation events in time. When studied at separate time points the following patterns were observed: 53 spots were changed compared to control at 3 hours, 27 spots at 6 hours, 67 spots at 24 hours and 52 spots at 48 hours. Only protein kinase A (PKA) and mitogen-activated protein kinase (MAPK) activation differed from uninfected lungs at all time points studied. Of note, the MAPK spot (960) is a MAPK_group phosphorylation site, thus does not indicate which pathway is implicated.

### *S. pneumoniae* impact on signaling in the lung

**Figure 3:**
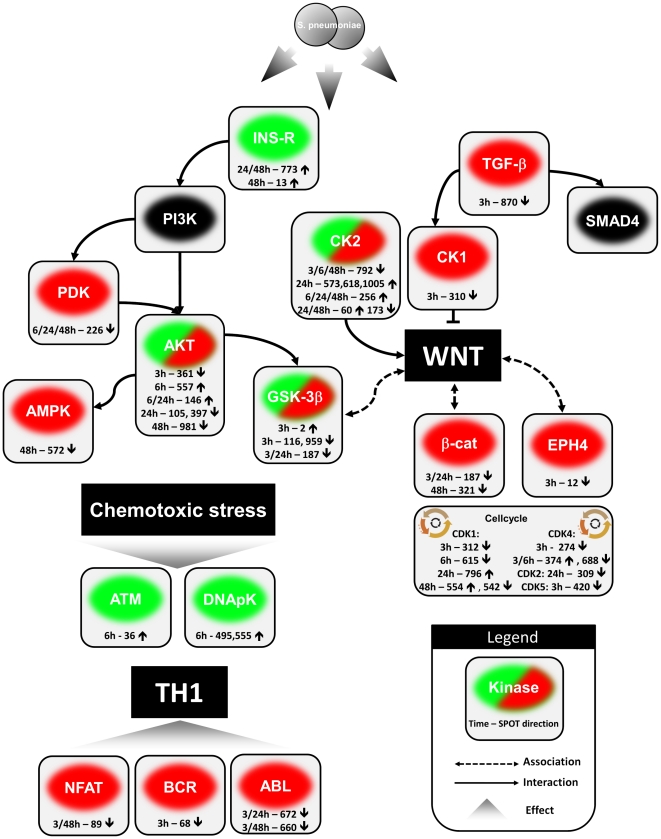
Provisional signal transduction scheme of active signaling pathways during pneumonia. Activation is depicted in green and inhibition in red, direction of events is calculated in relation to the noninfected control. Events from all timepoints (3, 6, 24 and 48 hours) were used to construct this scheme representing the entire host response dynamics of pneumococcal pneumonia. Important are the overall increment of chemotoxic stress and the initiation of the Th1 response. Spot numbers and activities (up ↑ or down ↓) are presented with corresponding kinases and timepoints. WNT signaling and the cell cycle are reduced throughout *S. pneumoniae* pneumonia.

Infection with *S. pneumoniae* induced a multitude of changes in kinome activity. Figure 3 shows an overview of ongoing processes during infection. Kinome chip analysis revealed that during pneumonia a Th1 associated signaling was induced as illustrated by the reduced activity of B-cell receptor (BCR, spot 68) at 3 hours, nuclear factor of activated T-cells (NFAT, spot 89) at 3 and 48 hours [19] and v-abl Abelson murine leukemia viral oncogene homolog 1 (ABL, spots 660 and 672) at 3, 24 and 48 hours [20]. Furthermore, increased activities of Ataxia telangiectasia mutated (ATM, spot 36) and DNA-dependent protein kinase (DNApK, spots 495 and 555) at 6 hours revealed the emergence of chemotoxic stress. ATM was upregulated likely due to presence of reactive oxygen species and DNA damage [21], [22]. DNApK activation occurs in response to DNA damage signals [23], [24].

In contrast to induction of chemotoxic stress, downstream insulin-receptor (INS-R) signaling was inhibited, as demonstrated by the reduced activity of pyruvate dehydrogenase kinase (PDK, spot 226) at 6, 24 and 48 hours, AMP-activated protein kinase (AMPK, spot 572) at 48 hours and the dynamic profiles of AKT (spots 46, 105, 361, 397, 557 and 981), which has events at all time points, and glycogen synthase kinase-3β (GSK-3β, spots 2, 116, 187 and 959) at 3 and 24 hours with both an increase and decrease in activity early during infection and a subsequent decrease/stabilization in the late phases of pneumococcal pneumonia.

A decrease in transforming growth factor- β (TGF- β, spot 870) activity was seen at 3 hours. Casein kinase-2 (CK2, spots 60, 173, 256, 573, 618, 792 and 1005) is involved and needed for WNT activation [25], while casein kinase-1 (CK1, spot 310) inhibits WNT signaling [26].

The found GSK-3β primary activation and reduction could also attribute to WNT signaling. In line, we found overall reduced activity of ephrin (EHP4, spot 12) at 3 hours and β-catenin(spots 187 and 321) at 3, 24 and 48 hours.

Interestingly, kinome array analysis revealed reduced activity for numeral kinases involved in regulation of the cell cycle (Cyclin-dependent kinase (CDK)1: spots 105, 3112, 542, 796, 554, 615/CDK2 spot 309/CDK4 spots: 274, 374, 688/CDK5 spot 420).

### Western blots of selected kinases confirm kinomics chip kinome profile data

**Figure 4:**
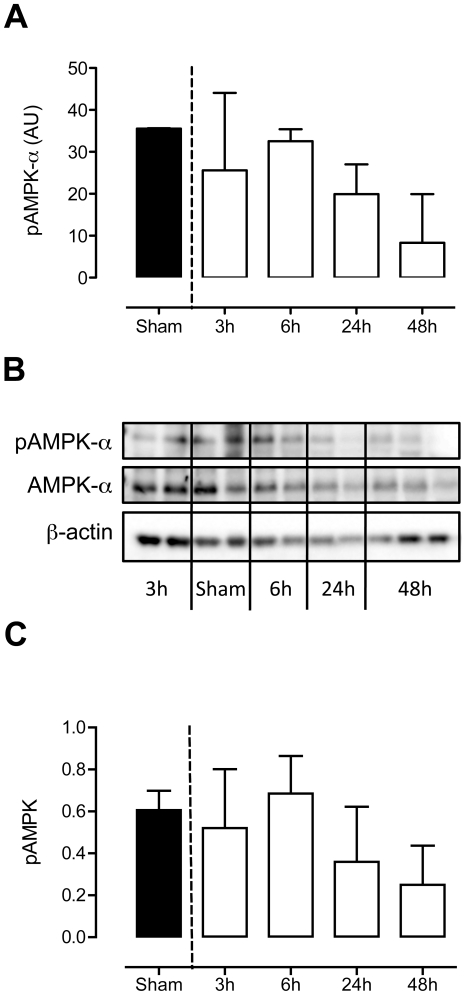
AMPK-α activity declines during pneumococcal pneumonia. Western blot analysis of phosphorylated AMPK-α (pAMPKα and unphosphorylated AMPK-α (b,c). During course of infection the ratio of pAMPK/AMPK decreased. A similar activity pattern was found in the kinomics chip (a). The bar graph shows quantification of the relative amounts of phospho-AMPK-α corrected for total AMPK-α. Data are presented as mean ± SD of n = 3.

In order to validate the phosphorylation-states of AMPK-α both phosphorylated and unphosphorylated AMPK-α were detected in lung homogenates from *S. pneumoniae* infected animals (Figure 4b). When comparing AMPK-α activity on the kinomics chip (Figure 4a) with the results from the western blots, a similar pattern emerged: decreased phosphorylation of AMPK-α at later time points of infection (Figure 4c).

**Figure 5:**
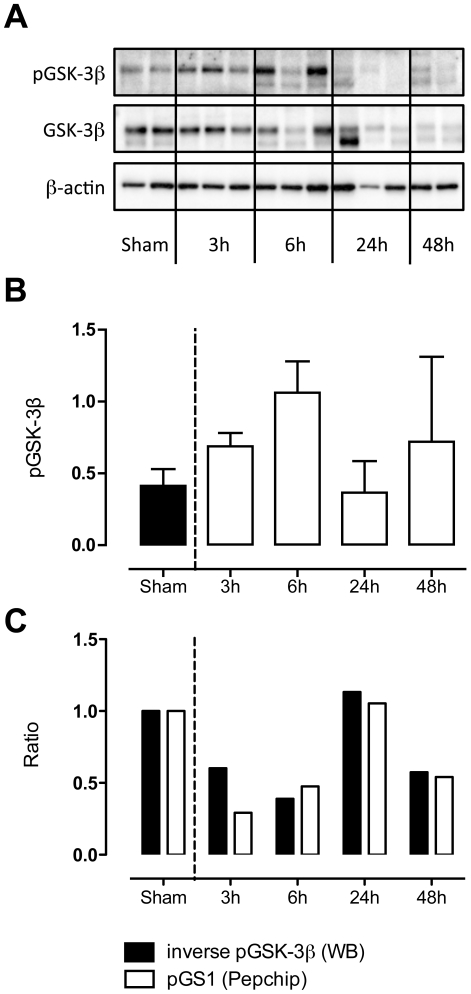
Early increase and later decrease in GSK-3β activity. Western blot of total GSK-3β and GSK-3β phosphorylation at ser9 (a: blot, b: quantification of phospho corrected for total GSK-3β). P-GSK-3β at Ser 9 inhibits phosphorylation of its substrate GS1 as depicted in Figure 5c. These data were derived from the kinomics chip. Data are presented as mean ± SD of n = 3.

To investigate if phosphorylation status of substrates on the kinomics chip mirrors that of more upstream kinases the phosphorylation state of Ser9, an inhibitory site of GSK-3β was measured. GSK-3β phosphorylates the substrate glycogen synthase 1 (GS-1). However inhibition of GSK-3β at Ser9 results in reduced GS-1 phosphorylation. Indeed, GS-1 on the kinomics chip showed reduced phosphorylation at all time points except for 24 hours after infection. As demonstrated by Figure 5a, GSK-3β activity was enhanced during the first 6 hours of infection. Activation pattern of the substrate GS-1 matched the activity of the upstream kinase closely: the ratio of the inverted p-GSK-3β signal closely resembled the ratio of the kinomics chip signal of GS-1.

**Figure 6:**
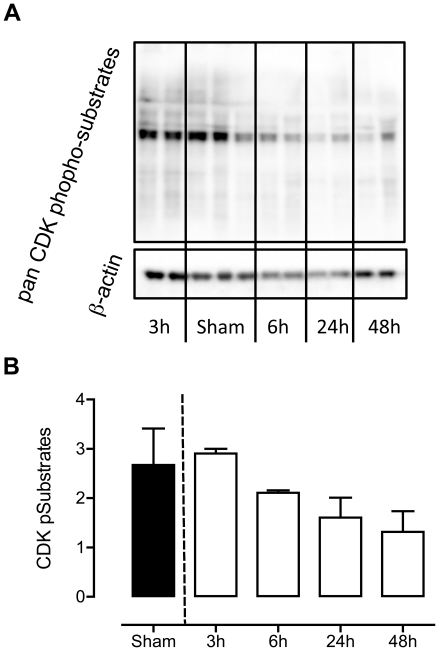
Decrease in CDK activity with increasing pneumonia. Western blot (a) and quantification of all CDK phosphorylated substrates (b) revealed a decrease in CDK activity. Quantification was performed for all positive signals on the blot and corrected for β-actin. Data are represented as mean ± SD of n = 3.

In general, CDK activity was decreased over time during pneumonia (Table 1). Since antibodies for the different CDK substrates (cyclins) are not available, we set out to validate these findings by using a pan CDK p-substrate western blot. By this approach we can detect activity of all CDKs at once. Semi-quantitative analysis of the blots validated the results obtained by the kinomics chip kinome profiles (Figure 6a, b): overall CDK activity in *S. pneumoniae* infection was decreased.

**Table 1:** Kinome chip analysis of cyclin-dependent kinase activity.

Kinase	Protein	Motif	Signal	Event time
CDK1	RAP1GAP	IVPGKSPTRKK	Down	24 h
CDK1	MYOD1	GDSDASSPRSN	Down	3 h
CDK1	CALD1	PTAAGTPNKET	Down	48 h
CDK5	DAB1	APRQSSPSKSS	Down	3 h
CDK5	MEF2A	KSEPISPPRDR	Up	24 h
CDK2-cyclin E	COIL	EAKRKSPKKKE	Down	24, 48 h

**Figure 7:**
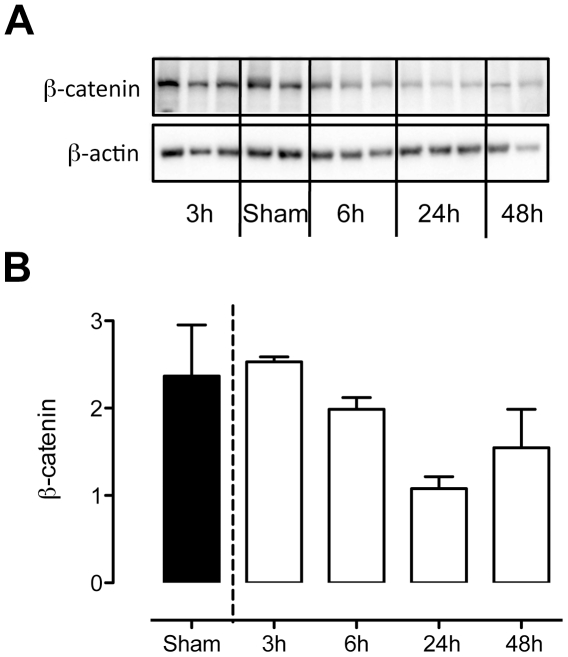
β-catenin levels decrease over time. Western blot (a) and quantification (b) of β-catenin. β-catenin protein levels decrease during *S. pneumoniae* pneumonia. Indirectly, represent a decrease of potentially active β-catenin. Data are represented as mean ± SD of n = 3.

Upon phosphorylation, β-catenin is rapidly degraded, reflecting the off state of WNT [27]. Unsuprisingly, only β-catenin levels were detectible by western blot assay (Figure 7). The total β-catenin amounts decreased over time. This, indirectly, represents a decrease of potentially active β-catenin.

## Discussion

Current mass spectrometry techniques and novel proteomics approaches like antibody microarrays determine protein phosphorylation levels rather than the enzymatic activity resulting from it, while measurement of kinase activity using the peptide microarray provides a direct view on the extent of enzymatic activity leading to specific signal transduction. We here utilized kinome substrate arrays to obtain insight in alterations in kinase activity in the lungs during respiratory tract infection caused by the most common causative agent in CAP, *S. pneumoniae*. The most strongly affected pathways identified during pneumococcal pneumonia were associated with enhanced chemotoxic stress, a developing Th1 response, reduced WNT signaling and down regulated cell cycle activity.

The infectious dose of *S. pneumoniae* chosen causes lethality in mice from 48 hours onward with an overall mortality rate of 90%–100% after 4 to 6 days [27], [28]. In accordance, fulminant pneumonia was induced, as demonstrated by a profound bacterial outgrowth and a strong induction of inflammatory cytokines and chemokines. We obtained lung samples between 3 and 48 hours after infection, thus presenting the entire dynamics of the host response during pneumococcal pneumonia from shortly after infection until shortly before death. As such, kinases activities found in the kinomics analysis are representative of severe pneumococcal pneumonia.

The kinome profile dendogram (Figure 2a) generated from lung samples harvested 6 hours after infection was more closely related to control than the kinome profile obtained after 3 hours. We hypothesize that this is the effect of early and rapid changes occurring in response to the introduction of the pathogen. Of note, cells migrating into lung tissue in response to *S. pneumoniae* entering the airways likely affect kinome profiling patterns, since these were generated from whole lung homogenates. Kinome studies on specific leukocyte subsets purified from lung tissue at several time points after induction of pneumonia might circumvent this shortcoming. However, cell isolation procedures potentially influence phosphorylation states and thus we chose to utilize total lung homogenates, generated from snap frozen material. One also has to consider that, when searching for new therapies, it is the whole lung that will be exposed to a potential drug, e.g. administered via nebulization. Therefore, we chose to determine kinase profiles of total lung lysates without isolating different cell types.

Analysis of our chip data revealed several significant ongoing processes in pneumonia. Much to our surprise specific MAPKs and other kinases classically related with the immune response, like those involved with TLR signaling (e.g. inhibitory κβ (IΚΚ)-α/IΚΚβ, TANK-binding kinase 1 and MAPK-kinase 1 (MEK-1)) were not prominently present in the results obtained from the kinomics chip, not even at the earliest time point. A general MAPK substrate however was shared between time points, but no links to its specific contexts could be made. Ex vivo stimulation of bone marrow derived macrophages with serotype 2 *S. pneumoniae* gave rise to p38MAPK/c-Jun N-terminal kinase and extracellular signal-regulated kinase (ERK) phosphorylation [30]. Moreover, in a murine model of pneumococcal pneumonia p38MAPK inhibition resulted in enhanced bacterial loads [31], thus indicating that MAPKs may play a role in pneumococcal pneumonia.

PKA was also shared in all time points. This kinase is involved in regulation of proliferation and differentiation, microtubule dynamics, chromatin condensation and decondensation, nuclear envelope disassembly and reassembly, as well as regulation of intracellular transport mechanisms and ion fluxes [32]. Thus, its overall presence is not surprising.

In context of an immune response to bacterial infection, the Th1 response signaling was the only prominent pathway that appeared in our analysis. Th1 responses were mirrored by the decreased activity of BCR and NFAT. De-phosphorylation of NFAT (as a substrate) enables its translocation to the nucleus inducing IL-2 transcription [33]. Contrary to NFAT and BCR, ABL activation was decreased reducing its Th1 inducing responses [34]. Although in this model of acute respiratory tract infection, the innate immune response plays an important role, T cell immune function is important for generating an adaptive immune response and memory building.

Moreover, in recent studies CD8 knockout mice or CD8^+^ T-cell depleted wild type mice displayed an increased susceptibility to serotype 3 pneumococcal pneumonia, while adoptive transfer of CD8^+^ T-cell to knockout mice improved survival [35]. Our Th1 results combined with this study demonstrate that T cells play a role in serotype 3 pneumoccocal pneumonia.

A variety of CDKs and CDK associated kinases displayed a reduced activity during the course of pneumococcal pneumonia. These kinases regulate the progression through the cell cycle [36]. However, recent studies demonstrate that utilizing a CDK inhibitor reduced cerebrospinal fluid leukocyte count, hemorrhagic events and improved recovery in a mouse model of antibiotic treated *S. pneumoniae* meningitis [37]. It is proposed that the inhibition of CDKs in effect facilitates induction of caspase dependent apoptosis via myeloid cell leukemia sequence 1 (MCL-1) [38], [39]. The functional role of CDKs during *S. pneumoniae* pneumonia remains to be established.

Several kinases not primarily associated with inflammation or infection, were abundant in our findings. Nonetheless, most of these kinases have been implicated to also contribute to an inflammatory response. AMPK-α and GSK-3β mainly are known in metabolic context, specifically in insulin and glucose signaling [40], [41], but also have strong connections with inflammation. AMPK activation results in reduced TLR4 dependent lung inflammation [42]. GSK3 inhibition reduced IL-12p40, IL-6 and TNF-α in a mouse model of tularemia[43]. Furthermore, TLR induced pro-inflammatory cytokine production was reduced in monocytes by GSK-3β inhibition [44]. TGF-β is known to have strong anti-inflammatory effects [45]. Both GSK-3β and TGF-β, via CK2, are implicated in WNT signaling [46]. Our kinome analysis demonstrated that WNT signaling is overall reduced during pneumonia. To our knowledge, the role of WNT signaling in pneumococcal pneumonia has not been clearly defined yet, although β-catenin signaling has been associated with MAPK-signaling and modulation of NFkB function [46], [47]. WNT5 was described to contribute to the inflammatory response of human macrophages[48].

It should be noted, however, that differences in pneumococcal serotype can illicit differential immune responses. Serotype 11a (M10) murine pneumococcal pneumonia resulted in an early local levels of TNF-α and IL-6 in the pulmonary compartment, whereas serotype 3 (ATCC6303) displayed a more delayed response, which remained until death occurred [49]. This serotype 3 data is accordance with induction of IL-6 observed here, which was detected after 24 hours of infection. Thus, the immune system seems to react in a delayed fashion to the serotype 3 pneumococcus.

Notably, kinome profiles were determined in homogenates prepared from right (whole) lungs. As such, since the extent of pneumonia and the associated inflammation likely are not equally distributed in all lung segments in our model of intranasal infection, our data are not representative for specific areas of infection. In addition, analyses of both (whole) lungs would have provided more definitive information about pulmonary kinome profiles, since this approach would have excluded bias due to unequal left-right distribution of the infection.

Here we demonstrate the use of a systems biology approach on kinome activity in the lung during pneumococcal pneumonia. We uncovered pathways that induce chemotoxic stress and promote the Th1 response. Moreover, we found an overall reduction in WNT signaling and reduction in cell cycle activity during the course of severe *S. pneumoniae* infection. These data may pave the way to future drug interventions seeking to interfere with specific signaling pathways e.g. activating WNT signaling or reducing CDK activity.

## Materials and Methods

### Ethics Statement

This study was carried out in accordance with the Dutch Experiment on Animals Act. The Animal Care and Use Committee of the University of Amsterdam approved all experiments (Permit number: DIX100121).

### Animals

For all experiments female C57Bl/6 mice (aged 10 weeks) were purchased from Charles River (Maastricht, The Netherlands). The Animal Care and Use Committee of the University of Amsterdam approved all experiments.

### Induction of pneumonia/inflammation

Pneumonia was induced as previously described [28], [29]. *S. pneumoniae* serotype 3 (ATCC 6303) was grown to a mid-logarithmic phase at 37°C in Todd-Hewitt broth supplemented with 0.5% Yeast extract (both Difco, Detroit, MI). Bacteria were harvested by centrifugation at 4000 rpm for 15 minutes; washed twice and resuspended in sterile saline at a concentration of 5×10^4^ colony forming units (CFU)/50 µl. Mice were inoculated with 50 µl of bacterial suspensions intranasally under isoflurane inhalation anesthesia (Upjohn, Ede, The Netherlands).

### Determination of bacterial load

Three, six, 24 and 48 hours after infection, 3 mice per time point were sacrificed by cardiac puncture under Domitor (Pfizer Animal Health Care, Capelle aan der IJssel, The Netherlands: active ingredient medetomidine) and Nimatek (Eurovet Animal Health, Bladel, The Netherlands, active ingredient ketamine) anesthesia. Left lungs were harvested and homogenized in 4 volumes of sterile saline with a tissue homogenizer (ProScience, Oxford, CT, USA). CFUs were determined from serial dilutions of samples, plated on blood agar plates and incubated at 37°C for 16 hours before colonies were counted.

### Kinomics chip profiling

Right lungs were snap frozen in liquid nitrogen, after which they were homogenized in three volumes of lysis buffer (MPER (Pierce, Rockville, WI, USA) enriched with 1 mM MgCl_2_, 1 mM -glycerophosphate, 1 mM Na_3_VO_4_, 1 mM NaF, 1 µg/ml leupeptin, 1 µg/ml aprotinin, and 1 mM phenylmethylsulphonyl fluoride). Homogenates were centrifuged at 14,000 RPM for 5 minutes and pellets were discarded. As a control, right lungs were harvested from 3 mice administered with sterile isotonic saline 3 hours earlier and treated as described above. For the kinomics chip kinome profile assay samples were pooled and diluted to a protein concentration of 1 mg/ml. Of these lysates, 80 µl was added to 12 µl of Activation mix (70 mM MgCl_2_, 70 mM MnCl_2_, 400 µg/ml PEG 8000 and 880 kBq [^33^P-γ]ATP) and this mixture was applied to a kinomics chip (Pepscan Presto, Lelystad, The Netherlands) per pool. The chips were incubated at 37°C for 2 hours. The arrays were washed twice in 2 M NaCl (1% TWEEN 20), once in PBS (1% SDS) and rinsed twice in demineralised H_2_O. Subsequently the chips were air-dried and exposed to a phosphor imager plate for 72 hours. Radioactive signals were measured using a phosphor imager (Storm™, Amersham Biosciences, Uppsala, Sweden).

### Analysis

For analysis, Spot density and individual background were analyzed using Scanalyse (http://rana.lbl.gov/EisenSoftware.htm). These data were exported to a spreadsheet (Microsoft, Redmond, WA, USA) for further analysis. Spot intensities were normalized to 90 percentile, as previously described [13], [50]. Non-significant data were not taken into account (assessed by students t-test). Hierarchical clustering was performed on normalized spot intensities as described by Johnson [18]. Distribution of shared events in time was visualized using Venny (Oliveros, J.C. (2007) VENNY http://bioinfogp.cnb.csic.es/tools/venny/index.html).

### Westernblot

Results from the kinome analysis were validated by performing phospho-specific Western blots for major kinases. Blots were done for: AMPK-α/pAMPK-α, GSK-3β/pGSK-3β, pan-CDK phospho-substrate, β-catenin (all antibodies from Cellsignaling Technology, Boston, MA, USA) and β-actin (Santacruz Biotechnology, Santa Cruz, CA, USA). Samples for western blotting were boiled at 95°C for 5 minutes in laemmli buffer and loaded onto SDS-PAGE gels. After electrophoresis the content of the gels was transferred onto Immobilon-PVDF membranes (Millipore, Billerica, MA, USA). The membranes were blocked in 5% BSA (Roche, Basel, Switzerland) in TBS-T at room temperature for 60 minute. All primary antibodies were diluted 1:500 with exception of β-actin, which was diluted 1:4000. The membranes were incubated overnight at 4°C. Next, the membranes were incubated for 60 minutes with 1:1000 anti-rabbit-HRP conjugated secondary antibody (Cell Signaling Technology) and blots were imaged using LumiLight Plus ECL (Roche, Basel, Swizerland) on a GeneGnome chemiluminescence imager (Syngene, Cambridge, UK).

### Cytokine and chemokine assays

For cytokine and chemokine measurements, left lungs were excised, weighted and homogenized in saline four volumes of saline. Homogenates were diluted 1:2 in lysis buffer (300 mM NaCl, 30 mM Tris, 2 mM MgCl_2_, 2 mM CaCl_2_, 2% Triton X-100, AEBSF(4-(2-aminoethyl)benzeensolfonyl fluoride, EDTA-Na_2_, 8 µg/ml pepstatin and leupeptin, pH 7.4) and incubated on ice for 30 minutes. Homogenates were centrifuges at 3600 rpm at 4°C for 10 minutes and stored at -20°C until use. IL-1β, IL-6, TNF-α, KC and MIP-2 were measured in lung homogenates using ELISAs (R&D Systems, Minneapolis, MN, USA) according to the manufacturer's instructions. Detection limits were: TNF-α: 62.5 pg/ml, MIP-2 and KC: 15 pg/ml and 31.25 pg/ml for IL-1β, IL-6.

## References

[1] van der Poll T, Opal SM (2009). Pathogenesis, treatment, and prevention of pneumococcal pneumonia.. LANCET.

[2] Mandell LA, Wunderink RG, Anzueto A, Bartlett JG, Campbell GD (2007). Infectious Diseases Society of America/American Thoracic Society consensus guidelines on the management of community-acquired pneumonia in adults.. Clin Infect Dis.

[3] Bartlett JG, Dowell SF, Mandell LA, File TM, Musher DM (2000). Practice Guidelines for the Management of Community-Acquired Pneumonia in Adults.. Clinical Infectious Diseases.

[4] Ho P-L, Cheng VC-C, Chu C-M (2009). Antibiotic Resistance in Community-Acquired Pneumonia Caused by Streptococcus pneumoniae, Methicillin-Resistant Staphylococcus aureus, and Acinetobacter baumannii.. Chest.

[5] Hanage WP, Fraser C, Tang J, Connor TR, Corander J (2009). Hyper-Recombination, Diversity, and Antibiotic Resistance in Pneumococcus.. Science.

[6] Yinnon AM, Butnaru A, Raveh D, Jerassy Z, Rudensky B (1996). Klebsiella bacteraemia: community versus nosocomial infection.. QJM.

[7] Akira S, Uematsu S, Takeuchi O (2006). Pathogen Recognition and Innate Immunity.. Cell.

[8] Knapp S, von Aulock S, Leendertse M, Haslinger I, Draing C (2008). Lipoteichoic Acid-Induced Lung Inflammation Depends on TLR2 and the Concerted Action of TLR4 and the Platelet-Activating Factor Receptor.. J Immunol.

[9] Dessing MC, Hirst RA, de Vos AF, van der Poll T (2009). Role of Toll-Like Receptors 2 and 4 in Pulmonary Inflammation and Injury Induced by Pneumolysin in Mice.. PLoS ONE.

[10] Ratner AJ, Aguilar JL, Shchepetov M, Lysenko ES, Weiser JN (2007). Nod1 mediates cytoplasmic sensing of combinations of extracellular bacteria.. Cellular Microbiology.

[11] Ishii KJ, Koyama S, Nakagawa A, Coban C, Akira S (2008). Host Innate Immune Receptors and Beyond: Making Sense of Microbial Infections.. Cell Host & Microbe.

[12] Diks SH, Kok K, O'Toole T, Hommes DW, van Dijken P (2004). Kinome Profiling for Studying Lipopolysaccharide Signal Transduction in Human Peripheral Blood Mononuclear Cells.. J Biol Chem.

[13] van Baal JWPM, Diks SH, Wanders RJA, Rygiel AM, Milano F (2006). Comparison of Kinome Profiles of Barrett's Esophagus with Normal Squamous Esophagus and Normal Gastric Cardia.. Cancer Res.

[14] Parikh K, Diks SH, Tuynman JHB, Verhaar A, Lowenberg M (2009). Comparison of Peptide Array Substrate Phosphorylation of c-Raf and Mitogen Activated Protein Kinase Kinase Kinase 8.. PLoS ONE.

[15] Zeng X, Tamai K, Doble B, Li S, Huang H (2005). A dual-kinase mechanism for Wnt co-receptor phosphorylation and activation.. Nature.

[16] Cadigan KM, Nusse R (1997). Wnt signaling: a common theme in animal development.. Genes & Development.

[17] Moon RT, Bowerman B, Boutros M, Perrimon N (2002). The Promise and Perils of Wnt Signaling Through β-Catenin.. Science.

[18] Johnson S (1967). Hierarchical clustering schemes.. Psychometrika.

[19] Hermann-Kleiter N, Baier G (2010). NFAT pulls the strings during CD4+ T helper cell effector functions.. Blood.

[20] Huang Y, Comiskey EO, Dupree RS, Li S, Koleske AJ (2008). The c-Abl tyrosine kinase regulates actin remodeling at the immune synapse.. Blood.

[21] Alexander A, Cai S-L, Kim J, Nanez A, Sahin M (2010). ATM signals to TSC2 in the cytoplasm to regulate mTORC1 in response to ROS.. Proceedings of the National Academy of Sciences.

[22] Khosravi R, Maya R, Gottlieb T, Oren M, Shiloh Y (1999). Rapid ATM-dependent phosphorylation of MDM2 precedes p53 accumulation in response to DNA damage.. Proceedings of the National Academy of Sciences of the United States of America.

[23] Kang MJ, Jung SM, Kim MJ, Bae JH, Kim HB (2008). DNA-dependent protein kinase is involved in heat shock protein-mediated accumulation of hypoxia-inducible factor-1alpha in hypoxic preconditioned HepG2 cells.. FEBS J.

[24] Sibanda BL, Chirgadze DY, Blundell TL (2010). Crystal structure of DNA-PKcs reveals a large open-ring cradle comprised of HEAT repeats.. Nature.

[25] Gao Y, Wang H-y (2006). Casein Kinase 2 Is Activated and Essential for Wnt/β-Catenin Signaling.. Journal of Biological Chemistry.

[26] Hammerlein A, Weiske J, Huber O (2005). A second protein kinase CK1-mediated step negatively regulates Wnt signalling by disrupting the lymphocyte enhancer factor-1/beta-catenin complex.. Cell Mol Life Sci.

[27] MacDonald BT, Tamai K, He X (2009). Wnt/beta-catenin signaling: components, mechanisms, and diseases.. Dev Cell.

[28] van Zoelen MAD, Schouten M, de Vos AF, Florquin S, Meijers JCM (2009). The Receptor for Advanced Glycation End Products Impairs Host Defense in Pneumococcal Pneumonia.. J Immunol.

[29] Dessing MC, Knapp S, Florquin S, de Vos AF, van der Poll T (2007). CD14 Facilitates Invasive Respiratory Tract Infection by Streptococcus pneumoniae.. American Journal of Respiratory and Critical Care Medicine.

[30] Ripoll VM, Kadioglu A, Cox R, Hume DA, Denny P (2009). Macrophages from BALB/c and CBA/Ca mice differ in their cellular responses to Streptococcus pneumoniae.. Journal of Leukocyte Biology.

[31] van den Blink B, Juffermans NP, ten Hove T, Schultz MJ, van Deventer SJH (2001). p38 Mitogen-Activated Protein Kinase Inhibition Increases Cytokine Release by Macrophages In Vitro and During Infection In Vivo.. The Journal of Immunology.

[32] Taskén K, Aandahl EM (2004). Localized Effects of cAMP Mediated by Distinct Routes of Protein Kinase A. Physiological Reviews.

[33] Riether C, Kavelaars A, Wirth T, Pacheco-Lûpez G, Doenlen R (2009). Stimulation of [beta]2-adrenergic receptors inhibits calcineurin activity in CD4+ T cells via PKA-AKAP interaction..

[34] Taskén K, Stokka AJ (2006). The molecular machinery for cAMP-dependent immunomodulation in T-cells.. Biochemical Society Transactions.

[35] Weber SE, Tian H, Pirofski L-a (2011). CD8+ Cells Enhance Resistance to Pulmonary Serotype 3 Streptococcus pneumoniae Infection in Mice.. The Journal of Immunology.

[36] Liu L, Schwartz B, Tsubota Y, Raines E, Kiyokawa H (2008). Cyclin-Dependent Kinase Inhibitors Block Leukocyte Adhesion and Migration.. J Immunol.

[37] Koedel U, Frankenberg T, Kirschnek S, Obermaier B, Häcker H (2009). Apoptosis Is Essential for Neutrophil Functional Shutdown and Determines Tissue Damage in Experimental Pneumococcal Meningitis.. PLoS Pathog.

[38] Raje N, Kumar S, Hideshima T, Roccaro A, Ishitsuka K (2005). Seliciclib (CYC202 or R-roscovitine), a small-molecule cyclin-dependent kinase inhibitor, mediates activity via down-regulation of Mcl-1 in multiple myeloma.. Blood.

[39] Duffin R, Leitch AE, Sheldrake TA, Hallett JM, Meyer C (2009). The CDK inhibitor, R-roscovitine, promotes eosinophil apoptosis by down-regulation of Mcl-1.. FEBS Letters.

[40] Rayasam GV, Tulasi VK, Sodhi R, Davis JA, Ray A (2009). Glycogen synthase kinase 3: more than a namesake.. Br J Pharmacol.

[41] Towler MC, Hardie DG (2007). AMP-Activated Protein Kinase in Metabolic Control and Insulin Signaling.. Circ Res.

[42] Yuskaitis CJ, Jope RS (2008). Glycogen synthase kinase-3 regulates microglial migration, inflammation, and inflammation-induced neurotoxicity..

[43] Zhang P, Katz J, Michalek SM (2009). Glycogen synthase kinase-3[beta] (GSK3[beta]) inhibition suppresses the inflammatory response to Francisella infection and protects against tularemia in mice.. Molecular Immunology.

[44] Jope R, Yuskaitis C, Beurel E (2007). Glycogen Synthase Kinase-3 (GSK3): Inflammation, Diseases, and Therapeutics.. Neurochemical Research.

[45] Taylor AW (2009). Review of the activation of TGF-{beta} in immunity.. J Leukoc Biol.

[46] Hu D, Bi X, Fang W, Han A, Yang W (2009). GSK3β Is Involved in JNK2-Mediated β-Catenin Inhibition.. PLoS ONE.

[47] Duan Y, Liao AP, Kuppireddi S, Ye Z, Ciancio MJ (2007). [beta]-Catenin activity negatively regulates bacteria-induced inflammation.. Lab Invest.

[48] Pereira C, Schaer DJ, Bachli EB, Kurrer MO, Schoedon G (2008). Wnt5A/CaMKII Signaling Contributes to the Inflammatory Response of Macrophages and Is a Target for the Antiinflammatory Action of Activated Protein C and Interleukin-10.. Arteriosclerosis, Thrombosis, and Vascular Biology.

[49] Ferreira DM, Moreno AT, Cianciarullo AM, Ho PL, Oliveira MLS (2009). Comparison of the pulmonary response against lethal and non-lethal intranasal challenges with two different pneumococcal strains.. Microbial Pathogenesis.

[50] Lowenberg M, Tuynman J, Scheffer M, Verhaar A, Vermeulen L (2006). Kinome Analysis Reveals Nongenomic Glucocorticoid Receptor-Dependent Inhibition of Insulin Signaling.. Endocrinology.

